# Striking Sentiments

**Published:** 1877-03

**Authors:** 


### STRIKING SENTIMENTS.

In June, 1842, I began the practice of transcribing any short impressive sentence which occurred in reading; this habit is commended to the young as possessing very great advantages, requiring at times considerable moral courage, but affording ever after an enduring source of instruction and enjoyment. The work began thus, with an occasional originality:

"In miscellaneous reading some sentences strike the mind with peculiar force. The following appear to me to be very truthful, and may possibly indicate character:"

Great devotion to literary pursuits, without active exertion, often engenders indecision and want of manliness.

The heart that has once unwisely confided may break in its loneliness, but can never trust wholly again.

The true education of each man should commence when his collegiate studies are concluded.

The happiness of the heart induces seriousness, not noise or mirth.

Love is the sun of every society of the moral universe.

The heart that has anything to love and is loved in return, can never be utterly and remedilessly wretched.

One may feel as solitary in a crowd as in a desert.

The remembrance of duties heartlessly performed gives little satisfaction.

It is easier to take away a good name than to restore it.

Better to have chosen friends in the country than to be chosen friends in town.

I scarcely ever knew an instance of the companions of one's boyhood being agreeable to the tastes of one's manhood.

A fool flatters himself, a wise man flatters a fool.

Moments sometimes make the hues in which years are colored.

Pride is a spring-board at one time, and a stumbling-block at another.

O, once I loved another girl!
Her name it was Mariar,
But Polly, dear, my love for you
Is forty-five times higher!

Few accomplishments so much aid the charms of female beauty as a graceful and even utterance, while nothing so soon produces the disenchantment that necessarily follows discrepancy between appearance and manner, as a mean intonation of voice or a vulgar use of words.

Men are seldom struck with incongruities in their own appearance any more than in their own conduct.

Tears to many bring relief, but to the broken heart they only widen the wound.

A dreary thing it is to walk through the crowded street and see smiles wreathing around bright faces when they meet faces as bright as themselves, glad eyes lighting up at the sight of those whom they love, friend meeting friend, taking him by the hand with kind wishes and inquiries, and then look in upon your own lonely heart and feel that none of these are for you.

Nothing is so apt to disgust a feeling mind as a mistaken zeal.

We may have a thousand resources of happiness, but without a consciousness of rectitude of purpose, not one of all of them will avail. Bolingbroke, impeached within twelve months for high treason both by whig and tory—whom he had alternately served—banished, confiscated, condemned, was found at last to make out happiness from a consciousness of his own designs, and to consider all the rest of mankind as uniting in a faction to oppress virtue, impersonated (as he imagined) in himself.

An ambitious mind can never be fairly subdued, but will still seek for those gratifications which retirement can never supply.

Things written on an occasion, seldom survive that occasion.

With what mingled pleasure and pride do we learn that some great man had some deficiency of which we ourselves feel conscious, then foolishly imagine that we are great from having one or more of the foibles of the great. They were great in spite of those foibles; we are great in consequence of them. Because Goldsmith was a fool in mixed company, and Addison an ass, it does not follow that all fools and asses are Goldsmiths and Addisons.

He who assumes to be what he is not, will inevitably become nothing at all.

If a man becomes notable, he must make, not receive, an impression.

The surest and most speedy road to distinction is the diligent cultivation of natural tact.

To be elucidated into obscurity.

There are hours in which the mind of a literary man is unhinged, when the intellectual faculties lose all their elasticity, and when none but the simplest actions are adapted to their enfeebled state. At such times Bayle looked at clowns, and the Jewish Socrates, Moses Mendelssohn, stood at his window and counted the shingles on his neighbor's house.

Bayle said he could never comprehend the demonstration of Euclid's first problem.

Reading without reflection will never make a man wise.

Invention depends on patience; contemplate your subject long; it will gradually unfold until a sort of electric spark for a moment convulses the brain and spreads down to the very heart a glow of irritation, then come the luxuries of genius.

Conscience, when once hushed to sleep, may rise to torture, but will wake no more to save.

We darken our own lot, then call our sorrows destiny.

Unruffled self-possession is the philosophy of society.

From the follies of youth spring many of life's after sorrows.

Every occurrence that awakens a new emotion in childhood, is the forerunner of everlasting consequences.

History, at best, is in many respects a fable, a record of mistakes and prejudices.

He who cannot conceal his vexation, makes himself a laughing-stock for his enemies.

